# Treatment decision-making and the form of risk communication: results of a factorial survey

**DOI:** 10.1186/1472-6947-4-20

**Published:** 2004-11-16

**Authors:** Larry A Hembroff, Margaret Holmes-Rovner, Celia E Wills

**Affiliations:** 1 Institute for Public Policy and Social Research, 321 Berkey Hall, Michigan State University, East Lansing, MI, 48824, USA; 2 College of Human Medicine, Michigan State University, East Lansing, MI, USA; 3 College of Nursing, Michigan State University, East Lansing, MI, USA

## Abstract

**Background:**

Prospective users of preventive therapies often must evaluate complex information about therapeutic risks and benefits. The purpose of this study was to evaluate the effect of relative and absolute risk information on patient decision-making in scenarios typical of health information for patients.

**Methods:**

Factorial experiments within a telephone survey of the Michigan adult, non-institutionalized, English-speaking population. Average interview lasted 23 minutes. Subjects and sample design: 952 randomly selected adults within a random-digit dial sample of Michigan households. Completion rate was 54.3%.

**Results:**

When presented hypothetical information regarding additional risks of breast cancer from a medication to prevent a bone disease, respondents reduced their willingness to recommend a female friend take the medication compared to the baseline rate (66.8% = yes). The decrease was significantly greater with relative risk information. Additional benefit information regarding preventing heart disease from the medication increased willingness to recommend the medication to a female friend relative to the baseline scenario, but did not differ between absolute and relative risk formats. When information about both increased risk of breast cancer and reduced risk of heart disease were provided, typical respondents appeared to make rational decisions consistent with Expected Utility Theory, but the information presentation format affected choices. Those 11% – 33% making decisions contrary to the medical indications were more likely to be Hispanic, older, more educated, smokers, and to have children in the home.

**Conclusions:**

In scenarios typical of health risk information, relative risk information led respondents to make non-normative decisions that were "corrected" when the frame used absolute risk information. This population sample made generally rational decisions when presented with absolute risk information, even in the context of a telephone interview requiring remembering rates given. The lack of effect of gender and race suggests that a standard strategy of presenting absolute risk information may improve patient decision-making.

## Background

In contemporary U.S. society, prospective users of preventive therapies often must evaluate complex information about therapeutic risks and benefits. While health information is increasingly available, it is frequently incomplete and sometimes inaccurate [1,2]. For people to be active participants in health care decisions, information regarding the benefits and risks of therapies must be presented in ways that support high-quality, effective decision-making [3,4].

Prior research on presentation formats that are most effective, comprehensible, and likely to lead to effective decision-making suggests that a critical element is the format of risk information [5]. Mazur [6] suggests that how patients use and react to information are important dimensions for assessing patients' understanding. For example, prior studies have demonstrated the importance of the visual aspects of information presented [7,8] on shaping preferences and use of information.

A key format difference is absolute versus relative gain or loss [9,10]. Absolute risk is a rate. It expresses the actual number of people experiencing an outcome, e.g., 1 in 1,000 women. Relative risk is a ratio (risk among exposed divided by the risk among the non-exposed). Thus, in relative risk terms, those who smoke are described as twice as likely as non-smokers to get lung cancer; taking medication X is described as cutting the risk of disease in half. However, relative risks do not reveal the actual rates of occurrence. Doubling a 2% risk is very different in its consequences from doubling a 40% risk. It has been previously demonstrated that relative risk presentation can produce errors. By "errors," we mean that people's perceptions of risk and benefit information does not conform to normative Expected Utility (EU) theory assumptions. Central to EU theory is the assumption that individuals who make "rational" choices should prefer an option with the highest expected utility relative to all options being considered.

Prior clinical studies have found that presenting information in absolute versus relative risk formats can affect the interpretation of information about medications and personal risks of developing diseases. For example, in a sample of family practice patients, Hux and Naylor [10] found that patients were less willing to take a lipid-lowering drug when risks were described in an absolute compared to a relative risk format (42% versus 88%). Likewise, in a relative risk assessment task, Woloshin et al. [11] found that about two-thirds of a sample of women who were actually at high risk for breast cancer classified themselves as being at less risk than they really were. However, results of clinical studies may or may not be generalizable to the general population. Without cross-sectional samples, it is difficult to develop a general population profile of those most likely not to understand health information, i.e., to not use it "correctly." This study tested the effects of alternative information presentation formats (absolute risk versus relative risk formats) on medication decision-making of adults in the general population.

## Methods

### Design

Data were collected as a part of a cross-sectional telephone survey of the adult, non-institutionalized resident population of Michigan. The telephone survey was part of a continuing series of quarterly surveys in Michigan known as the State of the State Survey (SOSS) conducted by Michigan State University's Institute for Public Policy and Social Research (IPPSR). 952 interviews were completed within the designated interviewing period. The margin of sampling error for the sample as a whole is ± 3.2%. The average interview lasted approximately 22.8 minutes (median = 22 minutes; standard deviation = 4.8 minutes). A more detailed description of the SOSS methodology, sample design, procedures, and content, is provided in Hembroff and Silver [12].

### Sample

The sampling design for the State of the State Surveys is a disproportionate stratified random sample of phone numbers across the state's regions, over-sampling the less populated regions, and the city of Detroit. Trained interviewers called the random-digit dial (RDD) sample of telephone numbers to identify those that were actually working household phone numbers. Within identified households, interviewers interviewed a single adult selected using the "next birthday" technique [13] in which the adult in the household who would have the next birthday was chosen to be the respondent.

The data being reported have been weighted to adjust appropriately for the unequal probabilities of respondent selection based on the number of phone lines to the household and the number of adults within the household. Cases were also weighted to adjust for differential response rates among categories of race, gender, age and to adjust for regional disproportionate sampling. The final weighted data file very closely matches the demographic profile of Michigan's adult population.

### Procedures and response rate

Interviews were conducted using the computer assisted telephone interviewing facility (CATI) of IPPSR's Office for Survey Research (OSR). Interviewing took place over a six week time period during June and July of 1997. The overall completion rate among eligible households was 54.3%. The calculated response rate is based on assigning final outcome dispositions codes according to Standard Definitions, the American Association of Public Opinion Research [14] guidelines for calculating response rates using Formula RR4.

### Presenting health chances and patient treatment decision-making

The interview contained a split-ballot experiment to examine the effects of alternative ways of communicating risks. All respondents were presented with a baseline scenario. In this case, we asked respondents to ...

"Suppose you had a friend who was told that she was very likely to get a bone disease that would gradually make her crippled. Suppose the doctor said that there was a medication she could take on a daily basis that would greatly reduce her chances of getting the bone disease."

Respondents were asked if they would recommend that their friend take or not take the medication. Respondents were then randomly assigned to be presented one of four follow-up questions. Each follow-up question provided additional information about the hypothetical medication in terms of its benefits or risks. Half of the respondents were told that the medication would increase the patient's risk of breast cancer while the other half were told that the medication would decrease the patient's risk of heart disease. For each half, however, the information was presented in one of two randomly assigned alternative formats. Regarding a decreased risk of heart disease, respondents were told either that:

"by taking the medication she would also reduce her risk of heart disease by more than half," or that

"by taking the medicine she would also reduce her risk of heart disease from 1 in 200 to 1 in 500."

Regarding the increased risk of breast cancer, respondents were told either that

"by taking the medication she would also double her risk of breast cancer," or that

"by taking the medication she would also increase her risk of breast cancer from 1 in 10,000 to 2 in 10,000."

Based on the additional information received, respondents were asked what they would recommend that their friend do. Subsequently, each of these four groups was randomly divided again and given the additional information about increased risk of breast cancer or decreased risk of heart disease that they had not yet received. Then they were asked one more time to indicate what they would recommend to the friend about taking the medication. Thus, respondents were asked to indicate their treatment recommendations three separate times, under three of nine sets of conditions represented by the table below.

An initial treatment recommendation was made based on the information given in condition 1, and then again based on the combinations of information represented by conditions 2, 3, 4, or 7. A third and final recommendation was made based on the combination of information represented in conditions 5, 6, 8, or 9. Comparing the results for conditions 2 through 9 to the results from the baseline in condition 1 allows an assessment of the incremental effect of each additional piece of information on the treatment recommendation.

The dependent variable is the decision to recommend the medication or not. We hypothesized responses to vary by risk format and number of competing risks. For example, the number of "don't know" responses would be greater in conditions where complete information about competing risks was given. The benefits-only format would produce a majority of positive recommendations. However, we could not predict how large those proportions might be. Direction of change was expected to be positive for added benefit information, negative for more risk information, and reflecting trade-offs when risks and benefits were present. That is, we expected respondents to make a "rational" choice based on the perceived greatest expected utility.

### Analyses

To assess whether additional risk information about breast cancer or heart disease affected decisions in conditions 2 through 9, we compared the distributions of change responses for each condition. A "non-change response" occurs when the respondent gives the same answer to the follow-up question as to the baseline question. All other patterns of answers, such as a "don't know" or a "don't take the medication response to the follow-up after giving a "take the medication" response to the baseline, or vice versa, constitute change responses. Although some error variance would be expected, analyzing the distributions of change responses across baseline decisions for the individual treatment conditions determines whether or not the experimental manipulations had any systematic effect.

If the added information supports rational decision-making, the percentage of "don't knows" and recommendations against taking the medication would go down if the net effect of the additional information is further risk reduction. Conversely, if the net effect of the additional information is to increase the risk of health problems, the percentage of change responses would be greater among those who initially said "don't know" or to take the medication. The greater the magnitude in the new absolute risk, the greater should be the percentage of respondents who should change their decisions. To test for the effect of risk information format, we aggregated the respondents' decisions. We dummy-coded whether the risk information had been presented in absolute risk terms or not. We also dummy-coded whether the information was given for breast cancer (= 1) or heart disease (= 0). To test the effect of format using logistic regression, we also aggregated recommendations. A dummy variable was created to indicate whether or not the risk information for breast cancer was presented in absolute risk terms (= 1), and similarly for heart disease risk.

To develop a profile of those who could not make a recommendation, we dummy-coded recommendations to the baseline scenario into those recommending either to take or to not take the medication (DONTKNOW = 0) and those who said they did not know what to recommend (= 1). To develop a profile of those who made seemingly non-rational recommendations, we dummy-coded recommendations to the baseline scenario into those recommending to take the medication (WRONG = 0) and those who recommended not to take the medication or said they did not know what to recommend (WRONG = 1).

All analyses were performed using SPSS for Windows 7.5.

## Results

A total of 952 individuals were interviewed. In the weighted datafile, 54% were female; 12% were African Americans and 3% were Other non-whites; 26% were under age 30, 40% were 30–49, 18% were 50–64, and 16% were 65 or older; and 43% had a high school education or less, 31% had some college, and 26% had a college degree or more education. The reported median household income was between $40,000 and $50,000. In terms of health indicators, 15% rated their health as only "fair" or "poor;" 14% reported engaging in no leisure time exercise; 25% were current smokers; 38% were overweight or obese; and 1% were heavy drinkers. This profile shows that the sample obtained was representative of the general population of Michigan. All demographic and clinical characteristics were within the survey's margin of sampling error for the prevalence rates for these reported by the Centers for Disease Control and Prevention (CDCP) for Michigan in 1997 except for leisure time exercise.

### Information-based decisions

In the baseline condition, two-thirds of the respondents (66.8%) said they would recommend their friend take the medication, one in nine (11.2%) said they did not know what to recommend, and 22.0% recommended that their friend not take the medication.

Table 2 shows the percentages of respondents who changed their recommendation under each of the eight additional information conditions from their baseline decision. For each column, only the percentage of respondents who changed is shown, along with the total number of cases who made each of the three possible baseline choices in that condition.

As shown in Table 2, the experimental manipulations were effective and influenced treatment decisions as predicted. The table shows that the additional information presented did significantly effect respondents' decisions in conditions 2, 3, 4 and 7. In conditions 2 and 3, with additional breast cancer risk information, initial positive or "don't know" recommendations were 4 to 6 times more likely to change with new information. In conditions 5 and 7, where new information was about reduced risk of heart disease, those who had initially recommended not taking the medication or did not know were 7 to 10 times more likely to change their decision than those who had already made positive recommendations.

Similarly, in conditions 5, 6, 8, and 9, the percentage of respondents who changed their recommendation from baseline differed. That is, the information in conditions 5, 6, 8 and 9 were similarly effective at creating systematic changes in respondents' recommendations. Furthermore, those who changed a response from "don't know" at baseline, made changes consistent with the health outcome logically effected. This was true across all eight non-baseline conditions.

### Format of risk presentation

Table 3 shows treatment recommendations for all nine conditions. Added information about reduced risk of heart disease increased the percentage of positive recommendations. The format appeared to make no difference, perhaps because the absolute risk of heart disease was roughly consistent with respondents' prior assumptions. However, the percentage of "don't knows" was greater under absolute risk conditions.

Table 3 demonstrates that the added information about increased risk of breast cancer dramatically reduced the percentage of positive recommendations. When presented only in relative risk terms, the percentage of positive recommendations declined by more than two-thirds. When increased risk was presented in absolute risk terms, and risks were very low, the decrease was smaller. The difference appears to be that, although doubled, the risk of breast cancer was still very small in absolute risk terms.

Table 3 shows the results for the conditions in which various combinations of additional information were given. Additional information that the medication would both double the risk of breast cancer and cut the risk of heart disease by more than half reversed decisions in the baseline condition. Virtually the same percentage of respondents chose to recommend against the medication as when only the additional breast cancer information was given. The change was much greater than when only the additional information about the reduced heart disease risk was given. On the other hand, when absolute risk for breast cancer increased and absolute risk of heart disease decreased, the percentage recommending medication was the same as the percentage recommending against medication. Both fall roughly midway between the corresponding relative risk percentages.

Where the increased absolute risk of breast cancer due to the medication was given along with information about the decreased relative risk of heart disease, decisions to recommend the medication were nearly as great as in the baseline condition. However, the evidence clearly suggests respondents paid attention to both additional pieces of information. Where the very low absolute risk of breast cancer was presented, the increased risk appeared to be more acceptable in the face of the benefit in reducing the risk of heart disease. Where respondents were given information in absolute risk terms for both breast cancer and heart disease, twice as many chose to recommend the medication as did where both risks were given only in relative terms. This was only two-thirds as many as did so in the baseline condition. Compared to the baseline condition, respondents seemed to use both additional pieces of information. This is consistent with Expected Utility theory predictions, but was clearest under absolute risk conditions.

Table 4 shows the overall results of risk format. The logistic regression equation predicted the correct outcome for 70% of the cases choosing to recommend not taking the medication and 68% of the cases recommending taking the medication, and 69% overall. The interaction term was significant, indicating that communicating the information in absolute risk terms significantly affected the chances that the respondents would elect to recommend taking the medication, but that the effect was different depending on the type of disease. Receiving the risk information in absolute risk terms for breast cancer significantly increased the likelihood of a positive recommendation, but was not so for heart disease. For heart disease, the information regarding the absolute risk of breast cancer appears to have suggested that the overall risk was lower than previously assumed. In this case, knowing the absolute risk made medication-induced risk more acceptable.

Table 5 shows the effect of absolute risk on decisions. Controlling for demographic, geographic, health, and health risk variables did not change the results (data not shown). The logistic regression equation predicted the correct outcome for 85% of the cases choosing not to recommend taking the medication, 36% of the cases recommending medication, and 69% overall.

The table (again) shows that the interaction term is significant, indicating that the effect of the risk information format for each disease differs depending on what is known about the other disease. In this case, the table indicates that communicating risk information about both heart disease and breast cancer in absolute risk terms significantly changes positive recommendations compared to scenarios in which respondents received risk information in other combinations of formats.

### Non-rational decisions or no decisions

A third of the cross-sectional sample chose something other than to recommend taking the medication in the baseline condition. While the primary purpose of this experiment was to explore the effect of information format on treatment decisions, additional analyses were performed to better understand individuals who seem to make decisions contrary to the evidence given, or who indicated an inability/unwillingess to make a decision. The two dummy variables constructed for this purpose, DONTKNOW and WRONG were analyzed against a broad set of demographic and clinical variables, including gender, race, ethnicity, living as a couple or not, age (in categories), rural/urban dwelling, education (in 4 categories), current smoker, current heavy drinker, perceived health as only fair or poor, physical activity, overweight, employed, and avoids medical care or uses alternative sources of care. (Heavy drinker and overweight definitions were taken from the Centers for Disease Control and Prevention (CDCP) Behavioral Risk Factor Surveillance System.

Table 6 indicates that those most likely to report not knowing what to do (DONTKNOW) were respondents who were not employed and those who had children under 18 living in the household. Other than these factors, none of the other variables were significant predictors. Those who were not working and those who had children at home under 18 were somewhat more likely than others to say they received most of their health information from newspapers or teachers. They were also somewhat less likely to say they received most of their information from doctors. Somewhat more than other respondents, these individuals also more frequently reported believing information in the media to be clear and not confusing. The number of cases on which this analysis is based is quite small (105 of the 937 respondents answered "don't know" to the baseline question) making the findings only suggestive. Nevertheless, it is interesting that those who were uncertain how to proceed were typically more likely to rely on newspapers and teachers rather than doctors as their major source of health information [17,18].

Table 6 also shows predictors of WRONG, i.e., those who gave either the "don't know" or "do not take the medication" responses. The table indicates that five variables were significant predictors. Hispanics were more likely to give the "non-rational" answer, as were those who smoke, older respondents, those with children living in the household, and those who had more education. Those who said their health was only "fair" or "poor" were actually more likely than others to give the "rational" answer.

## Conclusions

In general, the risk presentation format appeared to influence the resulting decisions. Without knowing the absolute risks, respondents appeared to assume the risk of breast cancer was much greater than the risk of heart disease, or that the perceived likely consequences of breast cancer were significantly less acceptable than those of heart disease. The pattern of recommendations was made to avoid the greater perceived risk. When the competing risks were stated in absolute risk terms, respondents changed their recommendations appreciably. The results of the experiment strongly suggest that people's prospective preventive treatment decisions can be greatly affected by the manner in which potential risks and benefits are communicated to them. When given information regarding the absolute risks associated with a proposed treatment, individuals make remarkably "rational" decisions, clearly indicating the weighing of risks and benefits.

It appears that presenting risks in absolute rather than relative risk formats may be more effective in helping respondents make decisions that are consistent with maximization of expected utility. It appears, in the scenarios devised here, this may be due to the previously documented finding that people exaggerate the risk of cancer [15-17]. They appear to draw on such preconceptions when phrases such as "double your risk" or "cut risk in half" are used in describing possible side effects of treatments. To some degree, exaggerated cancer risk perceptions may result from media reports, public health cancer screening campaigns, and defensive medicine protocols for screening for rare diseases [18].

Our findings seem to indicate that, in the absence of explicit information regarding the absolute risks of particular outcomes, patients interpret information about relative benefits or risks based on whatever preconceptions they may have about their chances of getting the disease in question. That is, they make reasonable use of the relative risk information if they do not know the base risk rates. However, to the extent that these preconceptions may be quite inaccurate, relative risk information may lead them to make decisions that seem irrational and inconsistent with their actual preference. Without information about the absolute risks, health information may become misleading and individuals may reach conclusions that go quite counter to what they actually think about the appropriate balance of benefits and risks of treatments.

Framing the scenarios as recommendations to a friend made the decision-making relevant to both males and females. The logistic regression analyses found no significant differences in decisions based on race, gender, or other demographic characteristics in this relatively large and representative sample. This suggests that these results may represent a general population response pattern. It is clear from the results that members of the public will generally use explicit absolute risk information in quite rational ways when it is available. Consequently, providers of health information, both public and clinical, would be well-advised wherever possible to provide patients with the necessary risk/benefit information in terms of the absolute risks associated with treatment options. Making the best information readily available and comprehensible may reduce reliance on errant preconceptions and opinions. This research suggests that having absolute risk information routinely available to patients and physicians for use in making clinical decisions may be an important part of improving health care decisions.

This study is unique in surveying the general public rather than clinical populations on the effects of risk formats on decision-making. However, it was limited to presenting risks only in terms of the chances of an outcome occurring. It did not address the magnitude of the outcome's impact on the individual's life or the acceptability to the individual of the relevant alternatives. Future research should address these aspects of risk as well as explicitly investigating assumptions about risks of specific diseases. Research that explicitly assesses general public risk perceptions may be valuable in determining the need for absolute risk information in specific disease contexts. Providing baseline absolute risk information in certain disease contexts may lead to improved patient treatment decision-making.

## Competing interests

The author(s) declare that they have no competing interests.

## Authors' contributions

LAH conceived and carried out the survey. MHR and CEW provided medical decision making context for interpretation and discussion. All authors read and approved the final manuscript.

## Pre-publication history

The pre-publication history for this paper can be accessed here:



## Figures and Tables

**Table 1:** Representation of the baseline and incremental information conditions of the decision experiment

**Medication will reduce friend's risk of crippling bone disease. And ...**		**Information given about heart disease risk**		
	
		None	Risk is reduced by more than half	Risk is reduced from 1 in 200 to1 in 500
**Information given about breast cancer risk**	None	1 Baseline Condition	4	7
	Risk Doubles	2	5	8
	Risk increases from 1 in 10,000 to 2 in 10,000	3	6	9

**Table 2:** Percentage of respondents who changed recommendation decision from baseline choice, by additional information condition

		Recommendation decision at baseline			
**CONDITION**^a^		Take medication	Do not take medication	Don't Know	χ^2^
2. BC doubles	% Changed	70.6%	12.5%	53.6%	56.74**
	(n)	(160)	(56)	(28)	
3. BC 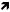 (1/10,000–2/10,000)	% Changed	34.7%	9.1%	59.3%	20.11**
	(n)	(144)	(44)	(27)	
4. HD < half	% Changed	5.8%	37.2%	51.7%	45.47**
	(n)	(137)	(43)	(29)	
7. HD 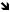 (1/200 – 1/500)	% Changed	4.5%	36.5%	28.6%	42.97**
	(n)	(177)	(63)	(21)	
5. BC doubles; HD < half	% Changed	67.6%	12.5%	25.0%	52.43**
	(n)	(136)	(56)	(16)	
6. BC 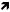 (1/10,000–2/10,000); HD < half	% Changed	19.1%	35.3%	64.9%	29.93**
	(n)	(141)	(34)	(37)	
8. BC doubles; HD 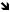 (1/200 – 1/500)	% Changed	58.0%	4.3%	58.6%	43.65**
	(n)	(181)	(46)	(29)	
9. BC 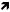 (1/10,000–2/10,000); HD 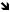 (1/200 – 1/500)	% Changed	42.8%	27.5%	54.5%	6.92*
	(n)	(159)	(69)	(22)	

* p < .05; ** p < .001

^a ^The numbers in the left margins of these rows refer to the condition numbers as identified in Table 1.

**Table 3:** % Recommending a friend take treatment for bone disease by nature of information given regarding effects on chances for breast cancer or heart disease

**Information given about ______ beyond baseline scenario**			Heart disease		
Breast cancer			None	Risk is reduced by more than half	Risk is reduced from 1 in 200 to 1 in 500

None	% who recommend	Take	66.8%	73.3%	71.6%
		Don't take	22.0%	15.2%	17.0%
		Don't know	11.2%	11.5%	11.5%
		(n)	(937)	(210)	(262)
Risk doubles	% who recommend	Take	21.2%	22.3%	31.3%
		Don't take	59.8%	60.8%	49.6%
		Don't know	19.0%	16.9%	19.1%
		(n)	(245)	(209)	(257)
Risk increases from 1 in 10,000 to 2 in 10,000	% who recommend	Take	49.0%	62.8%	42.9%
		Don't take	40.0%	26.1%	42.9%
		Don't know	11.0%	11.1%	14.2%
		(n)	(217)	(214)	(252)

**Table 4:** Results of Logistic Regressions of Recommending Taking Medication on Type of Disease and Format of Risk Communication (n = 930)

Independent Variable	Coefficient (B) (s.e.)	Exp(B)
Format Risk Communication (Absolute Risk = 1)	-.089 (.208)	.915
Type Disease (Breast Cancer = 1)	-2.326** (.221)	.098
Interaction	1.365** (.293)	3.914
Constant	1.012** (.156)	
Model χ^2^	179.781**	

**p < .001

**Table 5:** Results of logistic regressions of recommending taking medication on communicating breast cancer, heart disease risks in absolute terms (n = 926)

Independent Variable	Coefficient (B) (s.e.)	Exp(B)
Breast cancer (absolute risk = 1)	1.481** (.206)	4.397
Heart disease (absolute risk = 1)	.111 (.208)	1.117
Interaction	-.715** (.283)	.489
Constant	-1.075** (.152)	
Model χ^2^	75.102**	

**p < .001

**Table 6:** Results of logistic regression of "don't know" (DONTKNOW) and "not take the medication – don't know" (WRONG) answers on respondent demographic and health profile variables (n = 942)

	WRONG Answer		DONTKNOW Answer	
Variable	B	Exp(B)	B	Exp(B)
Sex	-.176	.839	-.188	.828
Race				
African Americans	-.408	.665	-.193	.824
Other non-white	-.938	.391	-.539	.584
Hispanic	.988*	2.687	-.546	.580
Health fair or poor	-.538*	.584	-.177	.838
No, only non-mainstream source health care	.155	1.167	-.637	.529
Married or living with partner	.126	1.134	-.017	.984
Where live				
Rural	-.054	.947	.242	1.274
Small town	-.303	.738	.426	1.531
Suburb	-.503	.605	-.235	.791
Currently employed	-.000	1.000	-.635*	.530
Current smoker	.445*	1.561	-.065	.937
Physically active	-.197	.821	.354	1.425
Heavy drinker	-.496	.609	.094	1.099
Age (in categories)	.152**	1.164	.129	1.138
Level of education (in categories)	.240**	1.273	.151	1.163
Children < 18 living in household	.795**	2.214	.714*	2.042
Currently overweight or obese	.062	1.064	.010	1.010
Constant	-1.973**		-3.271**	
Chi-square =		66.806**		29.727*
df =		18		18

* p < .05; ** p < .001
